# Body fluid levels of neuroactive amino acids in autism spectrum disorders: a review of the literature

**DOI:** 10.1007/s00726-016-2332-y

**Published:** 2016-09-29

**Authors:** Hui-Fei Zheng, Wen-Qiang Wang, Xin-Min Li, Gail Rauw, Glen B. Baker

**Affiliations:** 1 Mental Health Research Laboratory, Xiamen Xianyue Hospital, Xiamen, Fujian China; 2 Neurochemical Research Unit, Department of Psychiatry, Faculty of Medicine and Dentistry, University of Alberta, Edmonton, AB Canada

**Keywords:** Amino acids, Autism spectrum disorders, Glutamate, Glutamine, Taurine, GABA, Glycine, Tryptophan, d-Serine

## Abstract

A review of studies on the body fluid levels of neuroactive amino acids, including glutamate, glutamine, taurine, gamma-aminobutyric acid (GABA), glycine, tryptophan, d-serine, and others, in autism spectrum disorders (ASD) is given. The results reported in the literature are generally inconclusive and contradictory, but there has been considerable variation among the previous studies in terms of factors such as age, gender, number of subjects, intelligence quotient, and psychoactive medication being taken. Future studies should include simultaneous analyses of a large number of amino acids [including d-serine and branched-chain amino acids (BCAAs)] and standardization of the factors mentioned above. It may also be appropriate to use saliva sampling to detect amino acids in ASD patients in the future—this is noninvasive testing that can be done easily more frequently than other sampling, thus providing more dynamic monitoring.

## Autism spectrum disorders

The term autism spectrum disorders (ASD) refers to neurodevelopmental diseases that affect 1–2 % of children, according to the data on the broad array of ASD (Baron-Cohen et al. 2009). ASD is characterized by different levels of severity and occurs in all ethnic groups. Noto et al. (2014) reported that 1 out of 88 children aged 8 years will develop an ASD, with males more at risk than females. Blumberg et al. (2013) showed that the prevalence of ASD had risen 75 % from 2007 to 2012 in the United States. Feng et al. (2013) identified 12 studies when searching Chinese databases in 2013; the prevalence in these studies varied from 2.8 to 29.5/10,000.

ASD is characterized by impaired social interaction skills combined with restrictive/repetitive behaviors (American Psychiatric Association 2013). Genetic predisposition and environmental factors undoubtedly have effects on the pathophysiology of ASD, but the precise mechanisms related to the pathophysiology of ASD are unknown and definitive methods for prevention or treatment are lacking (Blaylock 2008). DSM-5 proposes that ASD symptoms must appear in the early childhood (infant) (American Psychiatric Association 2013). Receiving diagnosis at an early stage of development could contribute to the early intervention and therapy, benefiting both patients and their families (Zwaigenbaum et al. 2015; Sacrey et al. 2015; Brian et al. 2015). However, behavioral abnormalities are often overlooked in the early stage of ASD, even experienced professionals involved in pediatric healthcare (Howlin and Asgharian 1999). Therefore, many researchers have been trying to establish quantitative diagnostic criteria that could contribute to an early and more accurate ASD diagnosis. Many interacting factors are probably contributing to the etiology of ASD, and these potential factors are described in several excellent review articles (Lam et al. 2006; Pardo and Eberhart 2007; Aoki et al. 2012; Parellada et al. 2014; Lozano et al. 2015; Rozas et al. 2015; Subramanian et al. 2015; Zhang et al. 2015; Martin et al. 2016; Muller et al. 2016; Park et al. 2016); this review focuses on amino acids.

## Neuroactive amino acids

Several lines of evidence have shown that changes (e.g., availability, metabolism, and/or receptor activity) in neuroactive amino acids associated with central brain functions may play a role in the pathogenesis and/or pharmacotherapy of several psychiatric disorders (e.g., schizophrenia and mood disorders) that have symptoms, such as cognitive impairment and problems with social interactions, in common with ASD (Coyle 2006; Grant et al. 2006; Lam et al. 2006; Labrie et al. 2008; Ongür et al. 2008; Yüksel and Öngür 2010; Durrant and Heresco-Levy 2014). Several preclinical and clinical studies have implicated neuroactive amino acids in the etiology of ASD, fragile X syndrome, and tuberous sclerosis complex (TSC), but most of these studies have focused on glutamate, GABA, and/or glutamine (El-Ansary and Al-Ayadhi 2014; Rojas 2014; Santini et al. 2014; Rozas et al. 2015; Cochran et al. 2015; Lozano et al. 2015; Robertson et al. 2016). Other amino acids could also be involved and it may be important to conduct comprehensive studies in which a number of these amino acids are investigated simultaneously. Due to the potential role of neuroactive amino acids in the pathogenesis and treatment of ASD, monitoring changes in their concentrations in body fluids are also important in case they may be relevant to the early diagnosis and intervention in patients with ASD. This paper reviews the literature on such measurements of several of these important compounds in body fluids in ASD subjects.

## Glutamate (GLU)

GLU, which is highly concentrated in brain, is the primary excitatory neurotransmitter (Naaijen et al. 2015). GLU normally has a protective effect with regard to neural plasticity and cognitive function, but excessive GLU may be neurotoxic, leading to death of neurons and glia (Olney 1969; Manev et al. 1989) and may possibly play a role in the pathogenesis of psychiatric disorders, such as ASD (Sheldon and Robinson 2007). Ghanizadeh (2011) and El-Ansary and Al-Ayadhi (2014) showed that GLU is involved in neuroinflammation in ASD, and Ghanizadeh and Namazi (2010) proposed GLU and homocysteine as targets for therapy of ASD patients’ irritability and aggression. A hyperglutamatergic hypothesis of ASD has been proposed (Fatemi 2008; Blaylock and Strunecka 2009). Fatemi et al. (2002) showed that the levels of GAD 65 kDa and GAD 67 kDa proteins, both of which are involved in converting GLU to gamma-aminobutyric acid (GABA), are reduced in the brains of patients with ASD, resulting in the increased levels of GLU in the brain.

Studies on GLU levels in the plasma of patients with ASD compared to healthy controls (HCs) are contradictory, with some reporting increased levels (Moreno-Fuenmayor et al. 1996; Aldred et al. 2003; MacDermot et al. 2005; Shimmura et al. 2011; Tirouvanziam et al. 2011; Naushad et al. 2013; El-Ansary and Al-Ayadhi 2014; Cai et al. 2016) and some decreased levels (El-Ansary 2016). In addition, increased levels have been reported in serum (Shinohe et al. 2006) and decreased levels in platelets (Rolf et al. 1993) and urine samples (Evans et al. 2008; Yap et al. 2010; Nadal-Desbarats et al. 2014). Some studies have used magnetic resonance spectroscopy (MRS) to measure amino acid levels in patients with ASD and have reported that patients with ASD have increased GLU levels in brain (Page et al. 2006; Joshi et al. 2012; Hassan et al. 2013). Cochran et al. (2015) showed that, compared with HCs, patients with ASD had increased glutamine (GLN) levels, decreased GABA levels, and no difference in GLU levels in brain. However, van Elst et al. (2014) reported that GLU and GLN levels were decreased in ASD brains.

## Glutamine (GLN)

GLU is stored in the form of GLN in astrocytes until it is transferred to presynaptic terminals and converted back to GLU (Magistretti and Pellerin 1999). Ghanizadeh (2010) reported that a glutamine (GLN) synthetase inhibitor may improve inflammation in ASD. Shimmura et al. (2011) suggested that the level of GLN in plasma could be a screening test for detecting ASD in children, especially those with a normal intelligence quotient (IQ). In studies of GLN levels in ASD patients compared to HCs, plasma (Moreno-Fuenmayor et al. 1996; Aldred et al. 2003; Shimmura et al. 2011; Tirouvanziam et al. 2011; Good 2011a; Tu et al. 2012; El-Ansary 2016) and platelet (Rolf et al. 1993) levels have been reported to be decreased, serum levels to be no different (Shinohe et al. 2006), and urine levels either increased (Noto et al. 2014) or decreased (Evans et al. 2008).

## Taurine (TAUR)

TAUR is an osmoregulator and neuromodulator that suppresses vasopressin and has been reported to be depleted in urine of autistic children (Good 2011a). However, in other studies on TAUR levels in ASD patients compared to HCs, plasma levels have been reported to be increased (Moreno-Fuenmayor et al. 1996; Shimmura et al. 2011; Kuwabara et al. 2013) or decreased (Geier et al. 2009; Kern et al. 2011; Tu et al. 2012), and levels in urine samples to be increased (Yap et al. 2010; Nadal-Desbarats et al. 2014) or decreased (Ming et al. 2012). Although the results reported on TAUR levels in plasma and urine samples are contradictory, there is a consistent opinion that TAUR plays a protective role in patients with ASD (Good 2011a, b; Omura et al. 2015). Kuwabara et al. (2013) showed elevated plasma TAUR levels in adults with ASD and proposed that TAUR is compensatory against pathogenesis of ASD, such as that caused by oxidative stress.

## Gamma-aminobutyric acid (GABA)

The balance between GABA and GLU, inhibitory and excitatory neurotransmitters, respectively, is very important for brain function, and many psychiatric and neurological disorders may be the result of imbalance between GABA and GLU (Erickson et al. 2013; Rojas 2014; Robertson et al. 2016). Reduced GABAergic action in human and animal models of ASD has been proposed to be one of the reasons for an imbalance between excitation and inhibition (Rubenstein and Merzenich 2003; Gogolla et al. 2009; Blatt and Fatemi 2011; Ito 2016; Robertson et al. 2016). However, compared with HCs, GABA levels in plasma have been reported to be increased in ASD subjects (Dhossche et al. 2002; El-Ansary and Al-Ayadhi 2014). Dhossche et al. (2002) reported that plasma GABA levels tended to decrease with age in ASD. Compared with HCs, GABA levels in platelets have been reported to be decreased in ASD (Rolf et al. 1993) while those in urine samples increased (Cohen 2002). Neuroimaging techniques reported decreased GABA in brains of ASD patients (Kubas et al. 2012; Gaetz et al. 2014; Rojas et al. 2014; Omura et al. 2015; Cochran et al. 2015). Rojas et al. (2014) reported that the left perisylvian GABA levels were decreased in patients with ASD and their unaffected siblings. Recently, studies using oxytocin to treat animal models of ASD reported that oxytocin can increase excitatory GABA and enhance hyperglutamatergic activity (Tyzio et al. 2014; Young and Barrett 2015).

## Glycine (GLY)

GABA and GLY are major inhibitory neurotransmitters in the central nervous system (CNS). They act on receptors coupled to chloride channels which play an important role in normal function of the CNS (Ito 2016). GABA and GLY depolarize membrane potentials, acting as excitatory neurotransmitters during early development (Wang et al. 2002; Yamada et al. 2004; Kaila et al. 2014). They shift from excitatory to inhibitory neurotransmitters at birth and in maturation, and if that does not happen it may result in neurological disorders, including ASD (Tyzio et al. 2006, 2014). However, in some parts of the brain, GLY acts as a coagonist at NMDA GLU receptors (Martina et al. 2003; Baptista and Varanda 2005; Kim et al. 2005; Basu et al. 2009), and it has been suggested that the GLY/d-serine site on the NMDA receptor could be a target for ASD therapy. Compared with HCs, GLY levels in plasma (Tirouvanziam et al. 2011) and serum (Shinohe et al. 2006) of ASD subjects have been reported to be unchanged, and levels in urine samples reported to be increased (Nadal-Desbarats et al. 2014; Noto et al. 2014) or decreased (Evans et al. 2008; Ming et al. 2012).

## Tryptophan (TRP)

Serotonin (5-hydroxtryptamine, 5-HT) is an important neurotransmitter, and TRP is the precursor of serotonin (Zhang et al. 2015). Dysfunction of serotonin systems are implicated in some forms of ASD, and may contribute to social interaction impairments (Lam et al. 2006; Rubin et al. 2013; Yang et al. 2014). Whole blood serotonin has been reported to be elevated in at least 25 % of ASD children (Muller et al. 2016). However, reducing TRP in the diet can impair social behavior in patients (McDougle et al. 1996) and mice (Zhang et al. 2015) and increasing TRP in the diet has been reported to improve social behavior in mice (Zhang et al. 2015). Compared with HCs, TRP levels in plasma have been reported to be decreased in ASD (Tirouvanziam et al. 2011; Tu et al. 2012; Naushad et al. 2013), while Noto et al. (2014) reported levels to be increased in urine samples and Kałużna-Czaplińska et al. (2014) reported them to be decreased.

## d-Serine

In the recent years, d-serine in the brain has been the subject of extensive research (Fuchs et al. 2005, 2011; Nunes et al. 2012; Billard 2015; Sacchi et al. 2016). d-Serine is an important amino acid in glutamatergic transmission (Fuchs et al. 2005) and is a potent coagonist at NMDA receptors in some mammalian brain areas and possibly involved in the pathogenesis of several psychiatric and neurological disorders, such as schizophrenia (Labrie et al. 2008; Nunes et al. 2012; Balu and Coyle 2015; Ozeki et al. 2016), bipolar disorder (Yamada et al. 2004; Young and Barrett 2015), depression (Hashimoto et al. 2015, 2016; Deutschenbaur et al. 2016), Alzheimer’s disease (Paula-Lima et al. 2013; Madeira et al. 2015), and addiction (D’Ascenzo et al. 2014; Seif et al. 2015; Liu et al. 2016). GLY has high affinity for extrasynaptic NMDA receptors, while d-serine has high affinity for synaptic NMDA receptors (Vizi et al. 2013).

There is a paucity of studies on body fluid levels of d-serine in ASD. In 2006, Shinohe et al. (2006) showed that d-serine and l-serine levels in serum were no different between adult patients with ASD and HCs. Comparing with HCs, Tirouvanziam et al. (2011) showed that combined serine levels in plasma were decreased in ASD. In studies on urine samples, Kałużna-Czaplińska et al. (2014) reported that l-serine levels were decreased in ASD, and Noto et al. (2014) reported that l-serine levels were increased, while Evans et al. (2008) and Ming et al. (2012) showed that the combined serine levels were decreased. In the studies in which combined serine levels were reported, the d-serine and l- serine were not measured separately.

## Other amino acids

The metabolism of homocysteine is associated closely with folic acid and Vitamin B12 (Ghanizadeh et al. 2012; Desai et al. 2016). Desai et al. (2016) showed that a lack of folic acid may be involved in the pathogenesis of ASD. Bala et al. (2016) reported low plasma levels of Vitamin B12 in ASD compared to values in HCs. James et al. (2004), and Bala et al. (2016) reported that the concentration of homocysteine in plasma with ASD patients is low. However, other studies showed that homocysteine levels were increased in ASD patients compared with HCs: Tu et al. (2012) reported levels in plasma, Ali et al. (2011) and Paşca et al. (2006) reported levels in serum, and Noto et al. (2014) and Puig-Alcaraz et al. (2015) reported levels in urine samples, and all were reported to be increased. Puig-Alcaraz et al. (2015) found that increased urinary levels of homocysteine correlated directly with the severity of deficit in communication skills in ASD.

Arginine is an essential precursor for the synthesis of proteins and nitric oxide, and it can spare GLN, detoxify ammonia, and increase brain blood flow (Good 2011a). Compared with HCs, arginine levels in plasma of ASD patients have been reported to be increased (Kuwabara et al. 2013) or no different (Tirouvanziam et al. 2011).

Table 1 shows the reported levels of neuroactive amino acids in patients with ASD in comparison with HCs and includes some other amino acids not mentioned previously in this review (leucine, lysine, citrulline, alanine, valine, isoleucine, threonine, proline, methionine, aspartate, asparagine, phenylalanine, tyrosine, and histidine). Leucine, isoleucine, and valine are all termed branched-chain amino acids (BCAAs) and share a transport system with large, neutral amino acids (LNAAs), such as tryptophan, tyrosine, and phenylalanine which are the precursors of the neurotransmitter amines 5-hydroxytryptamine (5-HT, serotonin) and the catecholamines (Fernstrom 2005). Arnold et al. (2003) reported that the level of the essential amino acids valine, leucine, phenylalanine, and lysine in ASD was 58 % compared to HCs. Although there is a paucity of studies on the levels of BCCAs in ASD, most of the studies report a reduction of BCAA levels in autistic subjects (see Table 1 and the references mentioned therein), suggesting that future research in this area is warranted.

**Table 1:** Reported comparisons of the levels of neuroactive amino acids in patients with ASD and healthy controls

Amino acids	Specimen	Status
Glutamate	Plasma	Increased (Moreno-Fuenmayor et al. 1996; Aldred et al. 2003; MacDermot et al. 2005; Shimmura et al. 2011; Tirouvanziam et al. 2011; Tu et al. 2012; Naushad et al. 2013; El-Ansary and Al-Ayadhi 2014; Cai et al. 2016)
		Decreased (El-Ansary 2016)
	Serum	Increased (Shinohe et al. 2006)
	Platelets	Decreased (Rolf et al. 1993)
	Urine	Decreased (Evans et al. 2008; Yap et al. 2010; Nadal-Desbarats et al. 2014)
	Neuroimaging (Brain)	Increased (Page et al. 2006; Joshi et al. 2012; Hassan et al. 2013)
		Decreased (van Elst et al. 2014)
		No difference (Cochran et al. 2015)
Glutamine	Plasma	Decreased (Moreno-Fuenmayor et al. 1996; Aldred et al. 2003; Shimmura et al. 2011; Tirouvanziam et al. 2011; Good 2011a; Tu et al. 2012; El-Ansary 2016)
	Serum	No difference (Shinohe et al. 2006)
	Platelets	Decreased (Rolf et al. 1993)
	Urine	Increased (Noto et al. 2014)
		Decreased (Evans et al. 2008)
	Neuroimaging (Brain)	Increased (Cochran et al. 2015)
		Decreased (van Elst et al. 2014)
Taurine	Plasma	Increased (Moreno-Fuenmayor et al. 1996; Shimmura et al. 2011; Kuwabara et al. 2013)
		Decreased (Geier et al. 2009; Kern et al. 2011; Tu et al. 2012)
	Urine	Increased (Yap et al. 2010; Nadal-Desbarats et al. 2014)
		Decreased (Ming et al. 2012)
GABA	Plasma	Increased (Dhossche et al. 2002; El-Ansary and Al-Ayadhi 2014)
	Platelets	Decreased (Rolf et al. 1993)
	Urine	Increased (Cohen 2002)
	Neuroimaging (Brain)	Decreased (Kubas et al. 2012; Gaetz et al. 2014; Rojas et al. 2014; Omura et al. 2015; Cochran et al. 2015)
Glycine	Plasma	No difference (Tirouvanziam et al. 2011)
	Serum	No difference (Shinohe et al. 2006)
	Urine	Increased (Nadal-Desbarats et al. 2014; Noto et al. 2014)
		Decreased (Evans et al. 2008; Ming et al. 2012)
Tryptophan	Plasma	Decreased (Tirouvanziam et al. 2011; Tu et al. 2012; Naushad et al. 2013)
	Urine	Increased (Noto et al. 2014)
		Decreased (Kałużna-Czaplińska et al. 2014)
d-Serine	Serum	No difference (Shinohe et al. 2006)
l-Serine	Serum	No difference (Shinohe et al. 2006)
	Urine	Increased (Noto et al. 2014)
		Decreased (Kałużna-Czaplińska et al. 2014)
Serine (D- and L-)	Plasma	Decreased (Tirouvanziam et al. 2011)
	Urine	Decreased (Evans et al. 2008; Ming et al. 2012)
Homocysteine	Plasma	Increased (Tu et al. 2012)
		Decreased (James et al. 2004)
	Serum	Increased (Paşca et al. 2006; Ali et al. 2011)
	Urine	Increased (Noto et al. 2014; Puig-Alcaraz et al. 2015)
Arginine	Plasma	Increased (Kuwabara et al. 2013)
		No difference (Tirouvanziam et al. 2011)
Leucine	Plasma	Decreased (Arnold et al. 2003; Tirouvanziam et al. 2011; Tu et al. 2012)
	Cerebrospinal fluid	Decreased (Perry et al. 1978)
	Urine	Decreased (Evans et al. 2008)
Lysine	Plasma	Increased (Aldred et al. 2003; Arnold et al. 2003; Tu et al. 2012)
		No difference (Tirouvanziam et al. 2011)
	Urine	Increased (Noto et al. 2014)
Citrulline	Plasma	Decreased (Tirouvanziam et al. 2011)
Alanine	Plasma	Increased (Aldred et al. 2003)
		No difference (Tirouvanziam et al. 2011)
	Urine	Increased (Nadal-Desbarats et al. 2014; Noto et al. 2014)
		Decreased (Evans et al. 2008; Ming et al. 2012)
Valine	Plasma	Decreased (Arnold et al. 2003; Tu et al. 2012)
		No difference (Tirouvanziam et al. 2011)
	Urine	Decreased (Evans et al. 2008)
Isoleucine	Plasma	Decreased (Tirouvanziam et al. 2011)
	Cerebrospinal fluid	Decreased (Perry et al. 1978)
	Urine	Decreased (Evans et al. 2008)
Threonine	Plasma	Decreased (Tirouvanziam et al. 2011; Bala et al. 2016)
	Urine	Decreased (Evans et al. 2008)
Proline	Plasma	No difference (Tirouvanziam et al. 2011)
	Urine	Decreased (Evans et al. 2008)
Methionine	Plasma	Increased (Arnold et al. 2003; Naushad et al. 2013) Decreased (Bala et al. 2016)
	Cerebrospinal fluid	Decreased (Perry et al. 1978)
	Urine	No difference (Puig-Alcaraz et al. 2015)
Aspartate	Plasma	Increased (Moreno et al. 1992)
		No difference (Tirouvanziam et al. 2011)
	Platelets	Decreased (Rolf et al. 1993)
	Urine	Decreased (Evans et al. 2008)
Asparagine	Plasma	Increased (Aldred et al. 2003; Naushad et al. 2013)
		Decreased (Moreno-Fuenmayor et al. 1996; Tirouvanziam et al. 2011)
	Urine	Decreased (Evans et al. 2008)
Phenylalanine	Plasma	Increased (Aldred et al. 2003)
		Decreased (Arnold et al. 2003; Tirouvanziam et al. 2011; Naushad et al. 2013)
	Urine	Increased (Noto et al. 2014)
		Decreased (Evans et al. 2008)
Tyrosine	Plasma	Increased (Aldred et al. 2003)
		Decreased (Tirouvanziam et al. 2011; Tu et al. 2012; Naushad et al. 2013)
	Urine	Increased (Noto et al. 2014)
		Decreased (Evans et al. 2008)
Histidine	Plasma Urine	Increased (Bala et al. 2016) Decreased (Evans et al. 2008; Ming et al. 2012; Nadal-Desbarats et al. 2014)

## Discussion

Overall, the results on amino acid levels in ASD reported in the literature are, with the possible exception of the BCAAs, inconclusive, and contradictory. Table 1 is a summary of reported differences between ASD patients and HCs in the levels of amino acids. BCAAs are essential amino acids that make up about 1/3 of muscle protein, and these deficiencies may affect muscle and connective tissue integrity in ASD subjects (Evans et al. 2008). It has been suggested that BCAA deficiencies may be related to poor nutrition due to unusual food preferences in ASD children (Arnold et al. 2003).

In studies on amino acids levels in ASD subjects reported in the literature, there has been considerable variation in terms of factors such as age, gender, number of subjects, IQ, and psychoactive medication being taken. Future studies could be improved by standardizing these factors and analyzing levels of several amino acids (including d-serine and BCAAs) simultaneously.

Recently, many studies have focused on saliva samples to detect cortisol which is a good indicator of stress pressure and behavior recovery in patients with ASD (Putnam et al. 2012; Tordjman et al. 2014; Abdulla and Hegde 2015; Edmiston et al. 2015). Because of the ease of collecting saliva, it is convenient for caregivers to help patients, even infants and toddlers (Putnam et al. 2012), to collect samples at home. There should be reduced emotional changes compared to collecting blood samples and thus possibly increased accuracy of results (Woods et al. 2008). Unfortunately, few studies on amino acids in saliva with ASD patients have been done. It may be useful to employ saliva sampling combined with standardized conditions as mentioned in the discussion above to detect amino acids in ASD patients routinely in the future—this is noninvasive testing that can be readily done more frequently than other sampling, thus providing more dynamic monitoring.
